# Post traumatic brain perfusion SPECT analysis using reconstructed ROI maps of radioactive microsphere derived cerebral blood flow and statistical parametric mapping

**DOI:** 10.1186/1471-2342-8-4

**Published:** 2008-02-29

**Authors:** Anthony J McGoron, Michael Capille, Michael F Georgiou, Pablo Sanchez, Juan Solano, Manuel Gonzalez-Brito, John W Kuluz

**Affiliations:** 1 Department of Biomedical Engineering, Florida International University, 10555 West Flagler Street, EC 2671, Miami FL, 33199, USA; 2 Department of Radiology, Division of Nuclear Medicine (D57), University of Miami, P.O Box 016960, Miami, FL 33101, USA; 3 Department of Pediatrics, Division of Critical Care Medicine (R-131), University of Miami, P.O Box 016960, Miami, FL 33101, USA

## Abstract

**Background:**

Assessment of cerebral blood flow (CBF) by SPECT could be important in the management of patients with severe traumatic brain injury (TBI) because changes in regional CBF can affect outcome by promoting edema formation and intracranial pressure elevation (with cerebral hyperemia), or by causing secondary ischemic injury including post-traumatic stroke. The purpose of this study was to establish an improved method for evaluating regional CBF changes after TBI in piglets.

**Methods:**

The focal effects of moderate traumatic brain injury (TBI) on cerebral blood flow (CBF) by SPECT cerebral blood perfusion (CBP) imaging in an animal model were investigated by parallelized statistical techniques. Regional CBF was measured by radioactive microspheres and by SPECT 2 hours after injury in sham-operated piglets versus those receiving severe TBI by fluid-percussion injury to the left parietal lobe. Qualitative SPECT CBP accuracy was assessed against reference radioactive microsphere regional CBF measurements by map reconstruction, registration and smoothing. Cerebral hypoperfusion in the test group was identified at the voxel level using statistical parametric mapping (SPM).

**Results:**

A significant area of hypoperfusion (P < 0.01) was found as a response to the TBI. Statistical mapping of the reference microsphere CBF data confirms a focal decrease found with SPECT and SPM.

**Conclusion:**

The suitability of SPM for application to the experimental model and ability to provide insight into CBF changes in response to traumatic injury was validated by the SPECT SPM result of a decrease in CBP at the left parietal region injury area of the test group. Further study and correlation of this characteristic lesion with long-term outcomes and auxiliary diagnostic modalities is critical to developing more effective critical care treatment guidelines and automated medical imaging processing techniques.

## Background

Acute traumatic brain injury (TBI) is a leading cause of death and disability in children in the United States and represents a widespread clinical as well as socioeconomic problem [1]. Effective management of patients with TBI is based in large part on accurate assessment of the severity of brain injury both in the trauma center and after admission to the intensive care unit. Advanced diagnostic techniques, such as nuclear medicine imaging, can detect focal changes in cerebral blood flow (CBF) after TBI [2]. If focal changes in CBF after TBI can be detected and acted upon in time to prevent their deleterious effects which lead to worsening of the primary injury, then long-term outcome could be improved. The purpose of this study was to establish an improved method for evaluating regional CBF (rCBF) changes after traumatic brain injury (TBI) in piglets.

Many nuclear medicine studies designed to assess the correlation between CBF and severity of TBI are based on Single Photon Emission Computed Tomography (SPECT) cerebral blood perfusion (CBP) imaging [2]. SPECT is a widely used clinical tool that has proven to be useful not only for TBI but also in additional applications including detection of intracerebral hematomas due to stroke [3] and movement disorders due to closed head injury [4]. This work centers on SPECT CBP based detection focal changes in CBF in a pediatric model of severe TBI in piglets and the various computational and statistical methods developed to validate the approach. Alternative modalities such as MR and CT reveal anatomical changes (i.e. swelling) of the cortex at the TBI site, however damage at the site is not always accompanied by morphological changes. The radioactive microspheres technique (RMT) is the gold standard for measuring CBF. Both RMT and SPECT methods were carried out simultaneously, with SPECT CBP findings validated based on measurements derived from RMT.

Validation of SPECT measurements requires a method to match, or register, the location of the tissue samples removed for microsphere analysis with their corresponding location in the SPECT volume [5]. Fundamental to the RMT is the explicit design of a map of tissue areas where blood flow is measured. These areas being studied are referred to as regions of interest (ROIs) with a large number of contiguous ROIs comprising an ROI map. In this work methods were developed to digitize and reconstruct subject specific ROI maps into volume-of-interest (VOI) maps and then perform manual 3-D registration of the digitized VOI maps to the SPECT images. Here, ROI will be used in reference to RMT measured rCBF and VOI will be used to refer to SPECT data and the regions drawn during brain dissection. Accurate registration of the VOI maps allowed both the validation of SPECT data and detection of focal lesions by observing SPECT VOIs with a marked decrease in perfusion. Intrasubject lesion severity was determined by comparing its flow with the homologous region in the contralateral hemisphere of the brain and calculating the percent decrease, similar to the asymmetry index employed by Rousseaux et al. [3]. A voxel, as opposed to VOI level approach suitable for SPECT CBP image interpretation is statistical parametric mapping (SPM), which is considered part of the broader field of statistical neuroimaging [6,7]. The SPM methodology has been applied successfully to a range of clinical functional brain studies including head injury [8], cognitive rehabilitation therapy [9] and executive brain area functioning after TBI [10]. Detection of focal hypoperfusion requires a statistical model implementation to operate on homologous voxels within and between experimental groups to identify significant differences. Inferences are then made from the test statistics associated with each voxel. In this work, SPM analysis was performed to detect and characterize CBP at the maximum precision afforded by the SPECT machinery and experimental conditions.

## Methods

Male piglets weighing 4.1 – 11.2 kg were randomly assigned to either a test (n = 11) or control (n = 8) group. All were pre-anesthetized with an intraperitoneal injection of 40 mg/kg pentobarbital followed by intravenous administration of 5 μg/kg/hr fentanyl anesthetic and 0.2 mg/kg/hr pancurioum paralytic. Intracranial pressure, aortic pressure, end tidal respiratory CO_2 _concentration and body temperature were continuously monitored. Lines were inserted for injection of radiopharmaceuticals and microspheres, sampling of arterial and venous blood composition and reference organ withdrawal. Both groups received reflection of the scalp, a 1.3 cm craniotomy over the left parietal cortex and removal of the dura mater. TBI was simulated on test subjects by fluid percussion injury (FPI) at the exposed cortex. Following injury the animal was transported to the Nuclear Medicine Department at Jackson Memorial Hospital while placed on a ventilator to maintain blood gas and end tidal CO_2 _concentrations at normal levels. Upon arrival the standard monitoring devices were reconnected. SPECT imaging began approximately 2 hours post injury with the microsphere injection for RMT immediately after. Identical protocols were followed for control and test subjects except that test subjects received TBI.

### Nuclear medicine SPECT

The SPECT camera used is a Philips-ADAC Vertex Plus Hybrid PET/SPECT (ADAC Laboratories, Milpitas, CA) with two 180° opposed rotating detectors with low energy high-resolution collimators and a 5/8" NaI scintillation crystal. The software and hardware for image reconstruction was a Philips-ADAC Pegasys (Sun OS) processing workstation for reconstruction of the SPECT image data. Approximately two hours after TBI, before injection of post-injury microspheres, brain blood perfusion SPECT imaging was performed using ^99m^Tc-ECD. Scanning was initiated ten minutes after 5 mCi intravenous injection. Projections were collected over 24 minutes with each detector rotating 180° to make a complete 360° revolution in an elliptical orbit with the piglet placed in the supine position. Image volumes were reconstructed using the Philips-ADAC filtered backprojection algorithm with axial smoothing and a Butterworth filter with an order of five and cutoff of 0.45. Image volumes consisted of 64 × 64 × 64 voxels each measuring 4.38 mm^3^. The detector distance ranged from 14 cm to 20 cm during rotation resulting in a spatial resolution FWHM (full width at half-max) of approximately 12 mm [11].

### Ex-vivo RMT rCBF and in-vivo SPECT CBP registration

Radioactive microspheres are small (15 μm) radiolabeled latex beads that lodge in the capillaries as they pass through the organ tissue [12]. Blood flow in any particular tissue is proportional to the radioactivity emitted from the microspheres trapped in the specimen. A 100 μCi microsphere solution (either Cerium, Scandium, Niobium or Strontium) was injected immediately after SPECT. Blood withdrawal was performed for 1.5 min at a rate of 1.9 mL/min from the arterial line by a syringe pump (Harvard Apparatus, Holliston, MA). Approximately five hours after injury the subject was euthanized with an intravenous injection of KCl (4 m Eq/kg). The brain was removed and preserved in formalin allowing for the decay of the ^99m^Tc-ECD radiopharmaceutical used during the SPECT study.

**Figure 1:**
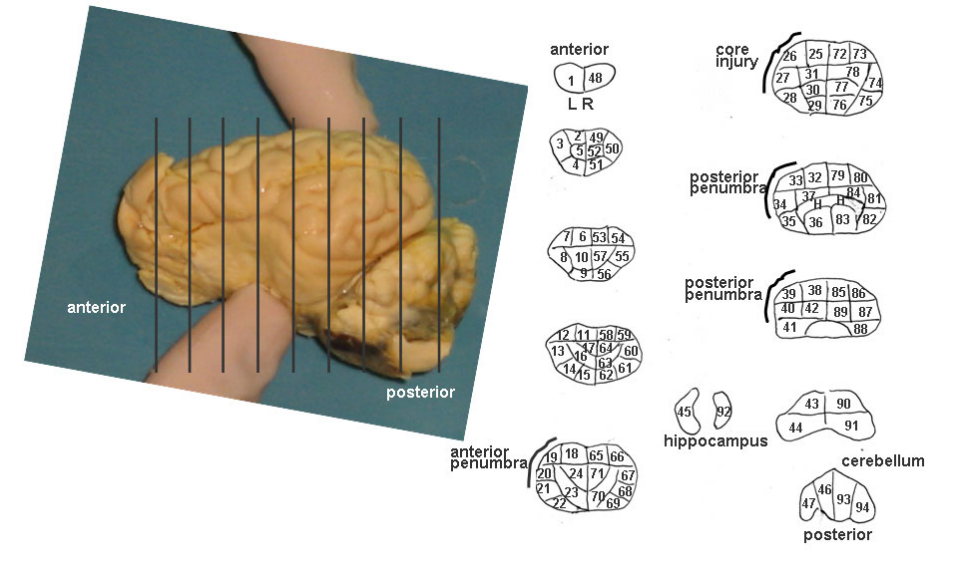
**Brain picture (left panel) and hierarchical dissection scheme example displayed as a CBF ROI Map (right panel).** A tracing was generated for each brain. The specific map for subject 10 is shown dissected according to the ROI template consisting of nine slices with 94 ROIs. Each slice is cut symmetrically left to right. The hippocampus (45, 92) and cerebellum (46, 47, 93, 94) are shown but were not included in the analysis. The remaining 88 ROI's were used in the analysis. The "core injury" region was identified as ROI's 25–27 based on visual examination after removal of the brain. The injury "penumbra" extended anterior and posterior to the core injury region as identified in the tracing.

A hierarchical dissection scheme, similar to that described by Gross et al [13], for the brain is shown in Figure 1. The right and left hemispheres were each carefully dissected into 47 regions of interest according to a standard grid (Figure 1). The cerebellum and hippocampus were not included in the analysis. Brain tissue samples weighed between 0.2 and 0.5 grams to ensure sufficient numbers of microspheres in each tissue sample. Generally, the dissection scheme can be thought of as a brain mapping template for each subject and will be referred to here as either the ROI template map, or template map. The ROI template map defined the number of serial slices and a series of samples contained within each slice. Two important features of the template map were: 1) minimal ROI size in order to detect sharp changes in rCBF and 2) ROI symmetry from the left to right hemisphere facilitating comparison of the injury site to the homologous region on the contralateral side. Dissection was performed manually, guided by the ROI template map resulting in a subject specific ROI map. Serial slices were made perpendicular to the longitudinal fissure and resulted in an average slice thickness of between 6 and 7 mm. Slices were then placed on heavy weight loose-leaf paper and further cut into symmetrical, left-right, samples following the map specification. After cutting, the outline of each sample was traced while in its natural position within the serial section. Finally, each sample was weighed and its radioactivity measured by a gamma-well counter (Packard, Minaxi Autogamma 5000).

Microsphere brain samples, similar to histological samples, are obtained by first fixing the specimen in tissue protecting alginate embedding material followed by slicing serial sections using a microtome. Successful reconstruction requires that the serial slices be realigned. Microsphere methods typically result in a relatively small number of slices whose manual realignment does not present such an onerous task. In this study, only nine slices were generated for each subject. Although the amount of realignment required was minimal, the small number of slices caused a loss of alignment information along the long axis of the brain. Registration of ROI map slices to target SPECT volume slices was achieved through visually guided manual placement in PMOD (PMOD Technologies, Ltd., Zurich, Switzerland) imaging software. The SPECT volume was translated, rotated and scaled in 3D space until a valid intermodality match was found for all slices in all dimensions. The piglet cerebrum was clearly located in the context of the head profile and reference anatomy points observable in the CBP image.

### Image volume manipulation and analysis

The PMOD (PMOD Technologies, Ltd., Zurich, Switzerland) medical imaging software, developed specifically for nuclear medicine studies was used. PMOD is a general tool for quantitative nuclear medicine image analysis and processing, and modeling of function and kinetics. Requirements for image volume viewing and manipulation and registration include volume loading, navigation and transformation. Viewing (PGATE) and VOI analysis (PVOI) tools are distinct modules operating under the main PMOD application. The PGATE viewer provided volume navigation simultaneously through the coronal, sagittal and transverse planes, or views. This method for displaying the brain slice images, orthogonal view, is essential for analyzing image volume data and is standard clinical practice. Orthogonal navigation works by first choosing a 2D location in one plane (e.g. transverse) and then displaying the orthogonal planes in the remaining views (e.g. coronal and sagittal). Affine geometric transformations were required to scale, rotate and translate the image volume for the ROI map registration process. PMOD applies the scaling transformation first followed by translation. Trilinear interpolation is used to interpolate pixel values when applying spatial transformations. Each *subject *image volume was spatially normalized to a *target *volume to ensure that the voxel intensity measurements originate from homologous locations in the different brains. Implementing spatial normalization requires considerations for resampling, smoothing, and geometric transformation.

### Statistical parametric mapping

VOI maps of SPECT perfusion cannot be statistically analyzed in a similar manner as the rCBF data (obtained from the RMT) due to the highly correlated nature of the measurements between adjacent SPECT VOIs. In order to analyze the injury groups for significant changes in rCBF a different strategy must be taken. As in ROI statistical mapping, a univariate test is performed at each data location, which is now an image voxel (counts) rather than an ROI region rCBF (mL/100 g/min). Simple correction methods for multiple comparisons between locations assume independence of the counts data at each voxel. As a result the correction is often too severe and statistically significant voxels are lost. The statistical parametric mapping technique as implemented by SPM2 (Wellcome Department of Imaging Neuroscience, UCL, UK) analyzes volumes for group effects at the voxel level and uses statistical theory to derive accurate estimates of data independence. Performing a statistical parametric mapping analysis requires three major stages: spatial image transformation, statistical model design, and setting parameters for making inferences on the model output.

### Focal TBI lesion detection

The methods outlined above were implemented to provide a framework for detecting focal TBI by measuring the spatial extent and magnitude of rCBF and CBP changes in control and injury groups. Initially, VOI maps were reconstructed from ROI maps to validate the accuracy of the SPECT CBP studies, demonstrating that CBP changes were correlated with, but not necessarily equal to, the location and magnitude of changes in the gold standard rCBF. Statistical parametric mapping was implemented to automatically detect focal changes in CBP based on differences between the groups and not *a priori *ROI definitions. Statistical ROI maps were created apart from SPM to validate the results. Computational tools used to perform the analyses were based on a combination of custom Matlab (Mathworks, Natick, MA) programming and image manipulation in PMOD. Statistical parametric mapping analysis was implemented through the SPM2 toolbox for Matlab.

### SPECT CBP: VOI map construction from ROI maps

ROI map slices were initially contrast enhanced, digitized and stored as jpeg image files. Digitization was performed by an HP Scanjet 3507c and HP Scanning software v1.0. Each serial section was scanned at a resolution of 100 dpi providing for both detailed resolution of ROI boundaries and a small file size. Output files were 276 × 276 pixels with 256 gray shades resulting in file sizes less than 100 KB. A total of nine jpeg files were created for each brain, one for each serial slice in the ROI map as in Figure 1. ROIs from the subject ROI maps, each with volume *v *and rCBF *x*, were reconstructed as volume-of-interest (VOI) maps with volume *v *and counts *y *(*R*_*i*_(*v*, *x*) → *V*_*i*_(*v*, *y*) *i *= 1.88) in 3-D space using the biomedical imaging software PMOD v2.5 (PMOD Technologies, Inc). Both the Gateway (PGATE) and VOI (PVOI) modules of PMOD were used extensively during reconstruction for affine image transformation and creation of VOIs, respectively.

**Figure 2:**
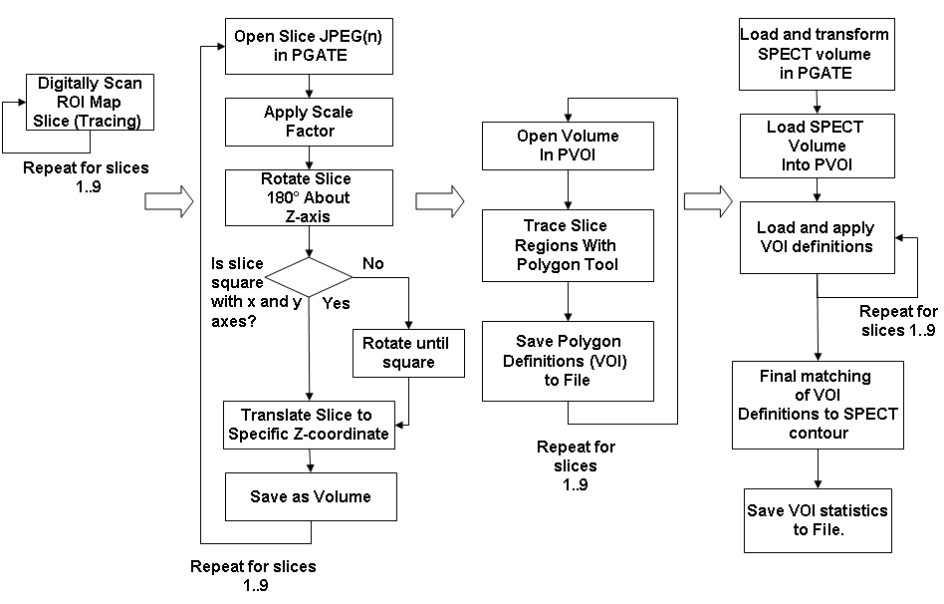
**Spatial 3-D reconstruction and registration to SPECT of the piglet brain ROI Maps were accomplished through the above process moving left to right in four phases and downward within each phase.** Each piglet brain consisted of nine slices and was processed individually.

Each ROI map slice was loaded (opened) as an image into PGATE as the first slice in a 276 × 276 × 64 image volume resulting in 63 blank slices plus the ROI map slice image. A flowchart (Figure 2) describes the procedure. This loading strategy allowed the slice to be translated and rotated 1) independent of the other ROI map slice images for the same brain and 2) in the same physical space as the SPECT volume. Each slice was loaded into PGATE individually and then the entire volume registration was verified. ROI map slice volumes were transformed to the same physical space as SPECT volumes by specifying the voxel dimensions as 1.02 mm × 1.02 mm × 4.38 mm and the origin coordinates. After loading, ROI slice image volumes were transformed to a common space. First, a scale factor of 0.37 was applied in the x and y dimensions to match the scaling of the SPECT volume. Next the slice was rotated 180° to match the supine positioning of the animal. Each ROI map slice image was then translated to a pre-specified z coordinate for that particular slice image. Any slices that were not originally drawn with midlines parallel to the x and y axes were corrected by rotation about its center. Following registration of each slice image volume to the common space, the volume was exported to PVOI for further processing. VOI slices were created by manually tracing individual ROI boundaries in the slice image. ROIs taken from the right hemisphere of the brain were traced first in numerical order and the process then repeated for the left hemisphere. After a VOI slice was created it was exported to a VOI definition file for later registration to the SPECT image volume.

### VOI map registration to SPECT image volumes

**Figure 3:**
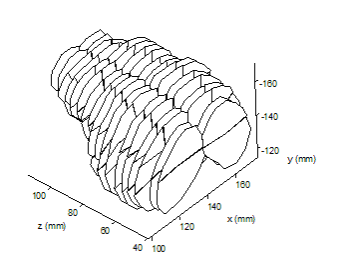
**VOI map of the piglet brain for subject 17 reconstructed from the subject's ROI map.** VOI map slices were duplicated to match thickness of scaled SPECT volume.

SPECT image volumes were loaded into PGATE for preprocessing. Volume scale was set to 1.5 in the x, y and z dimensions to adjust for the fractional slice thickness resulting from the brain dissection. ROI map slices were cut with a thickness between 6 mm and 7 mm, however the SPECT transaxial slice thickness was 4.38 mm. Scaling by 1.5 effectively decreased the SPECT slice thickness to 3.285 mm allowing assignment of two SPECT transaxial slices to one ROI map serial slice. The scale factor and 2:1 assignment scheme was fixed for all subjects. Following the scale operation, coarse affine transformations were performed on the image volume to align the subject's brain into the same common space as the reconstructed ROI maps. Complete VOI maps were then imported into PVOI along with the corrected SPECT image volume and the final transformations were made to visually align the VOIs to the contour of the brain in all three dimensions (Figure 3). Averaged counts, sum total counts and VOI volume (cm^3^) were calculated and stored for each VOI. Counts per gram for each VOI were obtained by dividing the sum total counts by VOI volume and multiplying by the inverse of cortex density (1.05 g/mL).

### SPECT CBP measurement accuracy

Both the reconstruction and registration components of the mapping methodology were tested for accuracy and precision using statistical analysis. Reconstruction tests examined the results of tracing ROI maps on paper and then in PVOI. SPECT CBP measurements are not absolute indications of blood flow, unlike microsphere rCBF, due to deterioration by three major sources of error: SPECT CBP imaging artifacts, map reconstruction, and map registration. Image artifacts include the physical effects of attenuation and scatter and smoothing caused by digital sampling (pixilation) of the field of view. Map reconstruction errors occur during excising, dissection, and tracing of the brain. When the brain is excised it tends to widen laterally and shorten vertically, although this effect is minimized by the formalin treatment. Lastly, map registration errors are introduced by the operator performing registration of the VOI map to the brain in the CBP images where suboptimal conditions, such as low uptake of the radiopharmaceutical tracer, can cause uncertainty over the correct organ landmarks in the image to align to the VOI map. Also, the VOI map must be aligned at points that are a multiple of the voxel size since the actual dissected brain slices did not have the same thickness as SPECT slices. For a large voxel size, this also may introduce error if the correct position for the VOI map is thought to be at a position in between two neighboring voxels, or planes. In examining the accuracy of the SPECT CBP data, the imaging, reconstruction and registration errors were considered collectively. The strength of the linear relationship between rCBF and CBP for each subject was tested using correlation analysis.

With the exception of extremely low or high flow values, rCBF and CBP are linearly related. Therefore, a suitable model for this relationship was the standard linear model of two variables. A predictive relationship between rCBF and CBP was established for each subject by fitting 88 paired ROI-VOI measurements to a line using linear regression analysis [12]. Both rCBF and CBP were normalized using the average value to a range between zero and one to compensate for the different units of measurement [12]. If the imaging, reconstruction and registration errors were minimal, the linear model would be a good predictor of CBP given rCBF (CBP′rCBF=c+0c1rCBF). This equation is used to compute predicted values of CBP, *CBP'*, given an independent value of rCBF and slope, *c*_1_, and intercept, *c*_0_. Values for *c*_1 _and *c*_0 _are derived using the experimental paired data points at each CBP-VOI and rCBF-ROI pair according to the standard least squares normal equations. From these linear regression calculations two important indicators of the rCBF and CBP relationship are obtained. A slope, *c*_1_, of 1 coupled with an intercept,*c*_0_, of 0 represents perfect agreement between rCBF and CBP values. Normally, these values will be less than optimal. The correlation coefficient, *r*, measures the degree to which there is a linear relationship between the two variables and always falls between – 1 and 1. Linear models and correlation coefficients were calculated for each subject and the entire population using the regress and corr2 functions in Matlab.

### Injury group lesion effect detection with SPM2

In the previous section the ROI Maps of rCBF values were analyzed for significant changes in rCBF due to TBI. The same analysis cannot be performed on the VOI maps derived from SPECT images because the CBP measurements are not independent and therefore no simple algorithm is available for adjusting the p value at each VOI for multiple comparisons. A statistical parametric mapping analysis, through SPM2, was employed to ultimately provide reliable estimates of independence and guidelines for inferring significant effects. Image volumes generated by the hybrid PET/SPECT camera stored in the raw data file format required conversion to the Analyze image file format for importation to SPM2. Subject 17 served as a template, or target, image to which all other subjects were spatially normalized. This particular subject was chosen because it was a control, had good contrast (high counts relative to background) and was ideally positioned in the gantry during the scanning process. Also, for optimal results the normalization algorithm requires that the selected template image have similar contrast to the image from which the parameters are to be determined. The contrast should be similar so that the normalization algorithm can reliably register the SPECT volumes based on perfusion values at each voxel.

The statistical model for comparing injured and sham groups was implemented as the predefined SPM SPECT model "*Compare Populations 1 scan/subject ANCoVA"*. This model performs voxel-wise unpaired t-tests between the control and test groups while taking the global CBP confounding effect into account using ANCOVA. Details of the SPM2 methods are described elsewhere [14]. The confounding effect, or nuisance variable in SPM2 parlance, is the background level of perfusion (counts/pixel) in the brain. The background level of perfusion was calculated as the right hemisphere average similarly to the ROI map analysis. All normalized images were loaded into SPM2 as belonging to either the test or control group. Each calculated subject covariate was entered as a single nuisance variable. ANCOVA was selected as the grand mean scaling method and no further grand mean scaling was used. The threshold for determining brain activity was relative and set, relying on general knowledge of the images, to 0.5. No sphericity correction was applied. Using the contrast manager GUI a contrast was implemented to specify the effect of interest as any significant decrease in the test group compared to the control group. ${\underline{c}}_{1}^{T}$ = (-1, 1). No Family-Wise Error (FWE) correction was applied in addition to the Gaussian random field correction due to the relatively small number of voxels being studied coupled with the low resolution of the SPECT CBP images. The uncorrected p-value voxel level threshold was set at 0.01 [15,16]. The extent threshold was set to three, thus avoiding spike activations, due to the small size of the brain and to more closely match the cluster size with the resolution of the SPECT camera,. This level is comparable to 5 reported in other studies using SPECT and fMRI [16,17].

## Results

### SPECT CBP accuracy

Overall, good agreement was seen between the SPECT CBP and microsphere rCBF data. Correlation coefficients, *r*, and their statistical significance, *p*, are listed in Table 1 along with the linear regression model for each subject. The total number of data points for each subject was 88. Due to the heightened sensitivity of the correlation test caused by large degrees of freedom, (p < 0.001) was considered significant. Comparisons based on unsmoothed rCBF measurements resulted in only two subjects obtaining high, (*r *= 0.521), and moderate, (*r *= 0.366), correlations and achieving statistical significance at (p < 0.001). The lowest correlation coefficients ranged from zero to negative. CBP and rCBF should increase or decrease in unison and since we could find no reason for negative values, they were not considered significant. The mean value for unsmoothed CBP-rCBF correlation, (0.056 ± 0.210), points to the absence of a linear relationship. Further, visual inspection reveals the relationship is virtually random. Clearly these results demonstrate a large amount of error compounded throughout the various processes culminating in SPECT CBP measurement. To compensate for this error the reconstructed rCBF volumes were smoothed with a Gaussian kernel of size 12 mm.

**Table 1:** Correlation coefficients (r), their associated p-values (p) and the computed linear regression for all subjects. Results based on unsmoothed rCBF values are in columns two through four while results based on smoothed rCBF reside in columns five through seven.

	Unsmoothed		Smoothed	
	
Subject	r	p	r	p
9	0.521	0.000	0.746	0.000
10	0.264	0.013	0.793	0.000
12	0.113	0.293	0.570	0.000
13	-0.118	0.272	0.224	0.036
14	0.366	0.000	0.758	0.000
15	0.156	0.146	0.479	0.000
16	0.001	0.992	0.370	0.000
17	-0.299	0.005	0.181	0.091
18	-0.289	0.006	0.386	0.000
21	0.179	0.096	0.700	0.000
22	-0.037	0.734	0.627	0.000
23	-0.068	0.531	0.650	0.000
25	0.118	0.273	0.555	0.000
26	0.021	0.849	0.523	0.000
28	0.098	0.366	0.345	0.001
29	-0.017	0.873	0.548	0.000
30	-0.051	0.639	0.599	0.000
31	-0.086	0.581	0.680	0.000
32	0.005	0.990	0.573	0.000
Overall	0.007	0.002	0.285	0.000
Corrected	0.014	0.000	0.344	0.000

**Figure 4:**
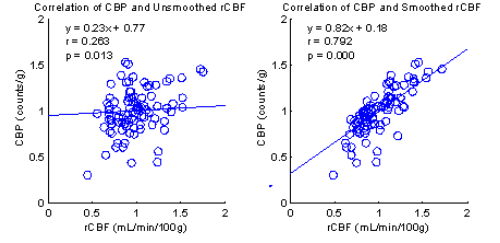
**Correlation data showing the effect of smoothing of the rCBF data for subject 10.** Smoothing accounts for a large portion of the major source of experimental error introduced by imaging artifacts and to a lesser degree by erroneous reconstruction and misregistration.

Comparing the right side of Table 1 with the left provides clear evidence that the smoothing of the rCBF measurements accounted for a large portion of the experimental error contributed by imaging artifacts and VOI map reconstruction and registration inaccuracies. The success of this smoothing approach can also be found in a similar study of multislice first-pass cardiac perfusion MRI by Epstein et al. [18]. The increase in accuracy is reflected by the higher correlation coefficients and slopes of the linear regression equations. This improvement is demonstrated in the mean *r *(0.532 ± 0.181), maximum *r *(0.793), and minimum *r *(0.181). No negative correlation coefficients survived the smoothing process. An example of the regression model improvement for subject 10 is shown in Figure 4. Recalling the major sources of error discussed earlier, it appears as though the rCBF smoothing succeeds by correctly compensating for the most damaging error source, SPECT CBP imaging artifacts. Imaging artifacts are caused by numerous sources including weak signals from low brain uptake of ^99m^Tc, camera insensitivity and low-resolution protocols. Through visual comparison of the corresponding smoothed rCBF slices and SPECT slices it was clear that the brain activity contours in all three dimensions co-registered reasonably well, which provided additional evidence supporting the validity of the approach.

### SPM

**Figure 5:**
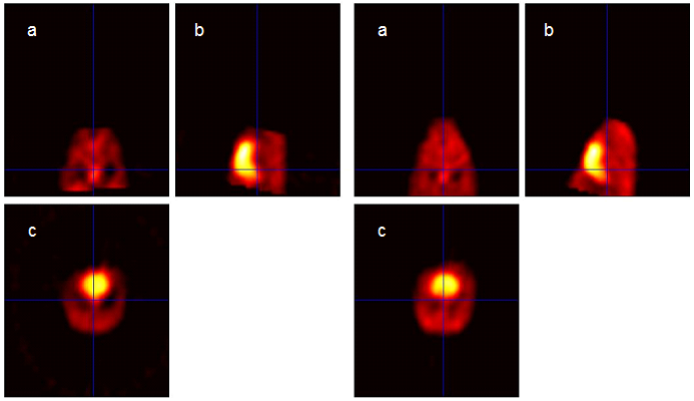
**Visual validation of spatial registration results was accomplished through side by side visual inspection using the SPM2 orthogonal viewer.** Orthogonal views are (a) coronal (b) sagittal and (c) transverse. On the left side are the othrogonal views for subject 16. On the right side are the three orthogonal views for the template, subject 17. The brain is shown as the area of highest intensity in views (b) and (c).

**Figure 6:**
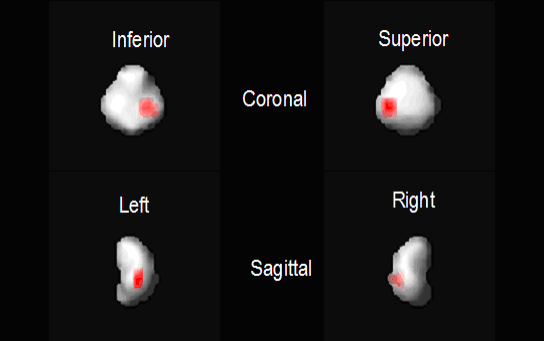
**Maximum intensity projections of significant decreases in SPECT CBP are rendered against a solid representation of the piglet brain.** The location of the focal decrease in SPECT CBP is consistent with the location of the induced left parietal injury.

Image data preprocessing consisted of spatial normalization between all subjects with subject 17 used as a template for registration. The error minimization algorithm was set to halt matching of each subject to the template at an error threshold of 0.05. Results of spatial normalization were determined to be successful by visual inspection using the orthogonal viewer in SPM2 (Figure 5). Model specification and parameter estimation in SPM2 resulted in a map of p-values for interpretation. The threshold t-score image in Figure 6 is given by the t contrast [-1 1] testing for any statistically significant decreases in CBP in the test group. The decrease of CBP in the left parietal region is clearly shown and is the only significant change reported in the brain area. The expected location of the lesion was identified by SPM for each of the 11 test animals (p < 0.01 for each case).

## Discussion

SPECT imaging is an important tool in assessing TBI [2]. Animal based pediatric models of TBI are especially suited for studying rCBF response to TBI by controlling the magnitude and location of the injury [19]. The rCBF gold standard RMT method used in animal models poses formidable visualization and registration challenges [5]. These challenges were overcome in this study through systematic application of a set of techniques. In order to visualize the ROI maps they were traced and reconstructed similarly to Gross et al [13] using a raster scanning algorithm proposed by Heckbert [20]. Registration of the ROI maps to the functional SPECT data did not require automated block matching algorithms as required by Ourselin et al. [21] because only nine slices per subject required registration in our application The visualization and registration process as a whole complements similar work such as registration of PET and ex vivo autoradiography data using manual matching of ROIs between modalities [22], registration of VOIs marked on digitized serial slice images and PET data using a maximization of mutual information metric between image pairs [23], and registration of serial slices to MRI by manipulating the position of the brain during sectioning [24]. We are aware of no studies dealing specifically with the registration of anatomical RMT derived ROI maps and nuclear medicine images.

Characterization of lesions induced by the TBI has been addressed in both animal models and in humans. SPM is becoming an increasingly popular approach in the field of statistical neuroimaging for interpreting lesions. Similar to clinical functional brain studies of head injury [8], SPM analysis was performed to detect and characterize CBP at the precision afforded by the SPECT camera and image volume reconstruction configurations. Although detection by SPM of the lesion induced through FPI was not surprising, valuable information was gained as to its spatial extent and relative magnitude. A drawback of the SPM approach to lesion detection is that multiple subjects for each treatment (i.e. injury) should be used in order to reach statistically significant areas of the image (e.g. a lesion). If the method were to be used in a clinical setting additional research may be needed to achieve high statistical power using a single subject. One approach to improving statistical power using a single patient is to compare a patient to a control group [8,25,26]. Statistical confidence can be improved by a large control group.

Characteristics of the lesion could be used to help build automated algorithms for the detection of TBI in pediatric patients. Existing algorithms designed to detect SPECT CBP asymmetries in a single adult subject use statistical techniques coupled with threshold comparison between the two hemispheres of the brain [27]. These algorithms are generally successful at finding asymmetries in the brain. However, whether the lesion is pathological in nature must be further studied by a clinician. Combining knowledge of the characteristics of this lesion from the study with automated searching could lead to a more powerful and useful medical image processing algorithm. Clinical applications notwithstanding, the SPM methodology as implemented here is unique in that no other studies using SPM to study functional data in an animal model were found to have been reported. The reconstruction, registration and smoothing techniques used to create the aggregate CBP-rCBF linear regression models can also be used for fluorescent microspheres studies such as by the one by Epstein et al. [18] and for arbitrary specimens, or organs. Future work needs to be done to automate the reconstruction and registration processes. It is anticipated that the tools developed here can be useful to other groups performing TBI research in animal models, or even more generally in research requiring map definition, registration and analysis. The linear regression of CBP with rCBF was not as strong as expected due to imaging artifacts largely because of the low resolution of the camera coupled with the small size of the piglet brain. Nonetheless, the model is useful for validation of the SPECT data. A limitation of this study is that the specific camera used is a hybrid for both SPECT and PET and is not optimized for either. Therefore, the spatial resolution is poorer than desired. Advancements in clinical SPECT imaging such as greater sensitivity, better spatial resolution and attenuation correction, may improve accuracy and detection by reducing partial volume effects and photon scatter.

## Conclusion

The objective of this work was to study the suitability of the application of SPM to an animal model of TBI and evaluate the correlation between the SPECT CBP and reconstructed RMT rCBF data. The suitability of SPM for application to the experimental model and ability to providing insight into CBF in response to traumatic injury was validated by the SPECT SPM result of a decrease in CBP at the left parietal region injury area of the test group. Regarding the potential for clinical SPECT CBP correlation with RMT rCBF, the aggregate linear regression model of CBP using rCBF was not as strong as expected due to SPECT imaging artifacts and the relatively small size of the piglet brain. Nonetheless, the model is useful for validation of the SPECT data. Advancements in clinical SPECT imaging such as greater sensitivity, resolution and attenuation correction, or use of a smaller detector tailored to animal research, would undoubtedly lead to improved accuracy and detection by minimizing artifacts such as partial volume effects and photon scatter while increasing sensitivity.

## Competing interests

The author(s) declare that they have no competing interests.

## Authors' contributions

AJM designed the study, and directed and coordinated all aspects of its performance. The paper was written primarily by AJM and MC. MC was responsible for image processing and data analysis. MFG coordinated the SPECT imaging laboratory and provided expertise in medical imaging. PS was responsible for conducting the SPECT imaging studies and for organizing the images with MFG for transfer to AJM and MC. JWK, JS and MB conducted the animal surgery and provided medical expertise in the brain injury model. All authors read and approved the final manuscript.

## Pre-publication history

The pre-publication history for this paper can be accessed here:


